# The YXXΦ motif within the severe acute respiratory syndrome coronavirus (SARS-CoV) 3a protein is crucial for its intracellular transport

**DOI:** 10.1186/1743-422X-11-75

**Published:** 2014-04-24

**Authors:** Rinki Minakshi, Kartika Padhan

**Affiliations:** 1 International Centre for Genetic Engineering and Biotechnology, New Delhi, India; 2 Laboratory of Systems Biology, National Institute of Allergy and Infectious Diseases, National Institutes of Health, Bethesda, MD 20892, USA

## Abstract

**Background:**

The SARS coronavirus (SARS-CoV) 3a protein functions as an ion channel, induces apoptosis and is important for viral pathogenesis. It is expressed on the cell surface and contains a tyrosine-based sorting motif and a di-acidic motif, which may be crucial for its intracellular trafficking. However the role of these motifs is not fully understood in the case of 3a protein.

**Methods:**

The subcellular distribution of the 3a protein was studied by immunofluorescence staining of cells transfected with wild type and mutant constructs along with markers for different intracellular compartments. Semi-quantitative RT-PCR was performed to estimate the mRNA where as western blotting was carried out to detect protein levels of wild type and mutant 3a proteins. In vitro transcription- translation was performed to estimate cell free protein synthesis.

**Results:**

While the wild type 3a protein is efficiently transported to the plasma membrane, the protein with mutations in the tyrosine and valine residues within the YXXV motif (ΔYXXΦ) accumulated in the Golgi compartment. However the 3a protein with mutations within the EXD di-acidic motif (ΔEXD) showed an intracellular distribution similar to the wild type protein. Increased retention of the ΔYXXΦ protein in the Golgi compartment also increased its association with lipid droplets. The ΔYXXΦ protein also expressed at significantly lower levels compared to the wild type 3a protein, which was reversed with Brefeldin A and Aprotinin.

**Conclusions:**

The data suggest that the YXXΦ motif of the SARS-CoV 3a protein is necessary for Golgi to plasma membrane transport, in the absence of which the protein is targeted to lysosomal degradation compartment via lipid droplets.

## Introduction

Severe Acute Respiratory Syndrome (SARS) originated in China towards the end of 2002 and quickly spread to about 30 countries by 2003 causing over 800 deaths worldwide [1-4]. The etiological agent of the disease, the SARS Coronavirus (SARS-CoV) was identified and classified as a unique member of beta Coronavirus. The SARS-CoV carries a ~30 kb positive-sense RNA genome that contains 8 unique open reading frames (ORFs) in addition to the common coronaviral genes [5,6]. The Orf3A is the largest among these ORFs and encodes a protein of 274 amino acids, the deletion of which reduces SARS-CoV replication in cell cultures and murine models of infection [7]. Antibodies against the 3a protein were also found in the sera of SARS patients [8]. Following infection of Vero E6 or CaCo2 cells, the 3a protein was found to be associated with virus particles [9,10] and localized to the plasma membrane and perinuclear regions of infected cells [11]. Several studies including ours have shown the 3a protein to induce apoptosis in host cell [12-14], which is also linked to its ability to form ion channels [15]. The 3a protein also promotes osteoclastogenesis by increasing NF-kB activity [16], and we have shown it to cause ER stress and induce down-regulation of the type 1 interferon receptor [14,17].

The 3a protein contains three transmembrane domains at the N-terminus and a C-terminal cytoplasmic domain of ~150 amino acids [18]. The cytoplasmic domain contains a tyrosine based sorting motif, YXXΦ (where X can be any residue and Φ is a residue with a bulky hydrophobic side chain) and a di-acidic EXD motif. It has been hypothesized that these motifs assist the 3a protein in regulating internalization of the viral Spike (S) protein from cell surface to intracellular sites [19]. In a study by Tan et al., the 3a protein lacking both of these motifs failed to express on the cell surface [11]. It has also been reported that the 3a protein is released in membranous structure from cell and mutation in the YXXΦ or EXD motif does not impact the release [20]. Tyrosine based sorting motifs are responsible for AP-2 mediated internalization from the cell surface by interacting with μ2 subunit of the clathrin complex [21]. However the YXXΦ motif can also mediate interaction with other members of the clathrin complex including AP-1, AP-3 and AP-4 for transport to different destinations inside the cell [22]. For example, the YXXΦ motif has been shown to be required for lysosomal targeting of some proteins like the CD3 chain of the T-cell receptor [23]. The di-acidic motif functions as a canonical ER export signal by mediating interaction with COPII vesicles [24-27] and has been shown to mediate efficient transport of the KAT1 ion channel protein to the plasma membrane [28]. However the functions of these individual motifs within the 3a protein have not been understood properly.

We previously reported the 3a protein to localize to the plasma membrane and to interact with Caveolin-1 [29], a protein that is part of lipid-rich regions of the membrane (caveolae) and has several functions in the cells, including the formation of lipid droplets and modulation of lipolysis [30-32]. Lipid droplets are intracellular storage organelles consisting of a core of neutral lipids surrounded by a monolayer of phospholipids [33]. Many proteins have been identified on lipid droplets that are also involved in vesicular transport, membrane fusion and cytoskeletal mobility. Among these are Perilipin A, Caveolins, Phospholipase D, and members of the Rab and ARF families of small GTPases [34-38]. Among viral proteins, the core proteins of hepatitis C virus (HCV) and GB virus B (GBV-B) are known to be associated with lipid droplets [39]. Recent views of lipid droplets emphasize that these might also act as storage vesicles for excess and unfolded proteins [34,40].

In this report we show that the tyrosine based sorting motif (YXXΦ) of the 3a protein is responsible for its sorting from the Golgi to plasma membrane. Whereas the wild type 3a protein traffics to plasma membrane efficiently, most of the YXXΦ mutants are retained in Golgi and lipid droplets. We also show that increased targeting of the 3a protein to lipid droplets is associated with its lysosomal degradation. These findings define the role of the YXXΦ motif in intracellular and surface transport of the SARS-CoV 3a protein.

## Results

### The YXXΦ motif of the 3a protein is required for its trafficking to the plasma membrane

**Figure 1:**
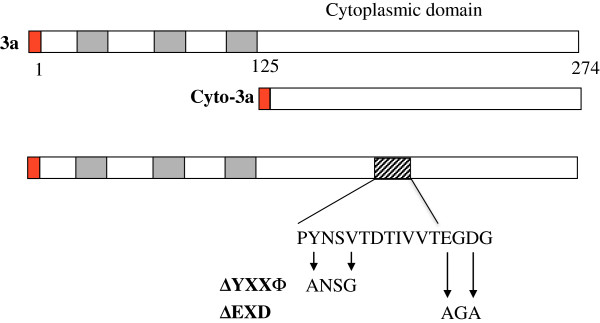
**The 3a protein domains and mutants.** The 3a protein has three transmembrane regions (grey boxes) between amino-acid 34–56, 77–99, 103–125, and YXXΦ and EXD motifs in the 150 amino-acids long cytoplasmic domain. The Cyto3a mutant lacks the three trans membrane regions. The amino acid changes in the ∆YXXΦ and ∆EXD mutants are indicated. An N terminal c-Myc epitope tag (red box) is attached to all the constructs.

**Figure 2:**
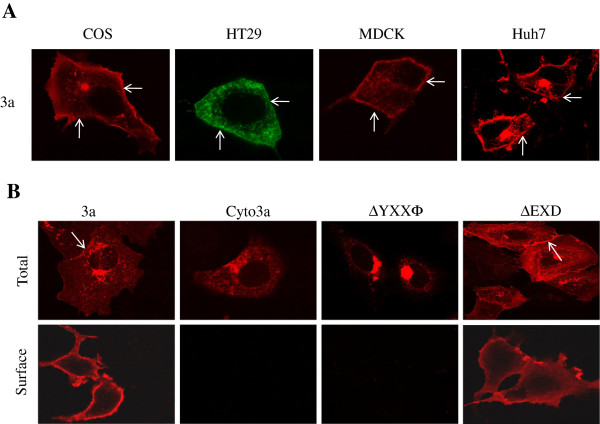
**Mutation of the tyrosine based sorting motif abrogates surface expression of the 3a protein. (A)** Distribution of 3a protein in different cell lines - COS-7, HT29, MDCK and Huh7. The pSGI-3A-HA construct was transfected into these cell lines and stained with rabbit anti-3a antibody and Alexa-594 conjugated anti-rabbit IgG. **(B)** Surface expression of 3a and its mutants - Huh7 cells were transfected with expression constructs for wild type or mutant 3a proteins. Protein expression on the cell surface was detected using an anti-Myc antibody without permeabilizing the cells; the total levels were detected with anti-3a antibody after permeabilizing the cells with methanol. Arrow indicates surface distribution. Data shown are representative of three different experiments.

Several motifs are present within the amino acid sequence of Orf3a that may be crucial for its intracellular localization. The 274 amino acids (aa) long 3a protein contains three potential transmembrane regions between residues 34–56, 77–99 and 103–125, followed by a cytoplasmic domain of ~150 aa (Figure 1). The cytoplasmic domain of 3a also contains a tyrosine-based sorting motif, YXXΦ (aa 160–163) and a di-acidic motif, EXD (aa 171–173). We compared the amino acid sequences of SARS-CoV 3a protein from several human and bat isolates available in NCBI database (Table 1). YXXΦ motif is conserved in all the isolates studied where as the EXD motif is mutated in all the seven bat isolates and one out of 21 human isolates. The EXD motif was proposed to be an endoplasmic reticulum (ER) export signal whereas the YXXΦ motif was shown to be crucial for endocytosis from the cell surface [19]. The topology of the 3a protein is such that the N-terminus is exposed to the outside of plasma membrane [11]. To study its cell surface distribution, we made expression constructs for the 3a protein and its mutants with a N-terminal Myc-tag. The 3a protein was expressed in four different cell lines – COS-7, HT29, MDCK and Huh7 and showed intracellular as well as surface localization in all cell types (Figure 2A). This result is consistent with previous reports showing the membrane distribution of the 3a protein [11].

**Table 1:** Comparison of amino acid sequences in the cytoplasmic tail of different SARS-CoV isolates from human and bat

**Isolates**	**Amino acid sequences (141-180) in the cytoplasmic tail of 3a**^**a**^
**Human Isolates**	
**ZJ0301**	YDANFVCWHTHNYDYCIP*YNSV*TDTIVVT*EGD*GISTPKL
**URBANI**	YDANFVCWHTHNYDYCIP*YNSV*TDTIVVT*EGD*GISTPKL
**TWJ**	YDANFVCWHTHNYDYCIP*YNSV*TDTIVVT*EGD*GISTPKL
**TWI**	YDANFVCWHTHNYDYCIP*YNSV*TDTIVVT*EGD*GISTPKL
**TOR2**	YDANFVCWHTHNYDYCIP*YNSV*TDTIVVT*EGD*GISTPKL
**Sino 3-11**	YDANFVCWHTHNYDYCIP*YNSV*TDTIVVT*EGD*GISTPKL
**Sino 1-11**	YDANFVCWHTHNYDYCIP*YNSV*TDTIVVT*EGD*GISTPKL
**SIN2774**	YDANFVCWHTHNYDYCIP*YNSV*TDTIVVT*EGD*GISTPKL
**SIN2748**	YDANFVCWHTHNYDYCIP*YNSV*TDTIVVT*EGD*GISTPKL
**SIN2679**	YDANFVCWHTHNYDYCIP*YNSV*TDTIVVT*EGD*GISTPKL
**SIN2677**	YDANFVCWHTHNYDYCIP*YNSV*TDTIVVT*EGD*GISTPKL
**SIN2500**	YDANFVCWHTHNYDYCIP*YNSV*TDTIVVT*EGD*GISTPKL
**LLJ-2004**	YDANFVCWHTHNYDYCIP*YNSV*TDTIVVT*EGD*GISTPKL
**HKU39849**	YDANFVCWHTHNYDYCIP*YNSV*TDTIVVT*EGD*GISTPKL
**GD01**	YDANFVCWHTHNYDYCIP*YNSV*TDTIVVT ***AGD*** GISTPKL
**CUHKU-W1**	YDANFVCWHTHNYDYCIP*YNSV*TDTIVVT*EGD*GISTPKL
**CFB/SZ/94/03**	YDANFVCWHTHNYDYCIP*YNSV*TDTIVVT*EGD*GISTPKL
**BJ04**	YDANFVCWHTHNYDYCIP*YNSV*TDTIVVT*EGD*GISTPKL
**BJ03**	YDANFVCWHTHNYDYCIP*YNSV*TDTIVVT*EGD*GISTPKL
**BJ02**	YDANFVCWHTHNYDYCIP*YNSV*TDTIVVT*EGD*GISTPKL
**BJ01**	YDANFVCWHTHNYDYCIP*YNSV*TDTIVVT*EGD*GISTPKL
**Bat Isolates:**	
**W1V1**	YDANFVCWHTHNYDYCIP*YNSV*TDTIVVT ***AGD*** GISTPKL
**RsSHC014**	YDANFVCWHTHNYDYCIP*YNSV*TDTIVVT ***AGD*** GISTPKL
**Rs3367**	YDANFVCWHTHNYDYCIP*YNSV*TDTIVVT ***AGD*** GISTPKL
**Rs672**	YDANFVCWHTHNYDYCIP*YNSV*TDTIVVT ***AGD*** GISTPKL
**Rf1**	YDANFVCWHTHNYDYCIP*YNSV*TDTIVVT ***SGD*** GISTPKL
**HKU3-12**	YDANFVCWHTHNYDYCIP*YNSV*TDTIVVT ***SGD*** GISTPKL
**279/2005**	YDANFVCWHTHNYDYCIP ***YNS I*** TDTIVVT ***SGD*** GISTPKL

^a^Amino acid sequences were obtained from National Center for Biotechnology Information (NCBI). The YXXΦ (where X is any amino acid and Φ is an amino acid with a bulky hydrophobic side chain) and EXD motifs are shown in italics whereas mutated motifs are highlighted in bold letters.

To study the subcellular distribution of the 3a protein we performed immunofluorescence staining of cells transfected with wild type and mutant constructs. The cell surface distribution was observed in non-permeabilized cells with the antibodies against the N-terminal Myc-tag. Staining of permeabilized cells with antibodies against the cytoplasmic region (aa 126–274) of the 3a protein was used to see its total expression and intracellular distribution. The wild type 3a protein was found to localize to the plasma membrane as well as to intracellular regions that appeared to be perinuclear and showed a punctate distribution (Figure 2B). The Cyto3a protein that lacks the transmembrane domains showed similar intracellular distribution, but was expectedly not found on the plasma membrane (Figure 2B). Surprisingly, the ΔYXXΦ protein with mutations in the tyrosine-based sorting motif was mainly localized in the perinuclear compartment (Figure 2B). On the other hand, the ΔEXD protein with the mutations in the diacidic motif distributed similar to the wild type protein. Since an N-terminal myc tag was present on all the proteins and was likely to be exposed extracellularly, staining non-permeabilized cells with the anti-myc antibodies confirmed the plasma membrane localization and proper topology of the 3a proteins. Only the wild type and ΔEXD proteins stained with anti-myc antibodies in non-permeabilized cells. These results showed that the 3a proteins are expressed with the correct topology of extracellular N-terminus and cytoplasmic C-terminus. The ΔYXXΦ mutant protein saturated in the intracellular compartment, but was not found at the plasma membrane, showing this tyrosine-based sorting motif to be important for its trafficking to the cell surface. The di-acidic motif, which is a canonical ER export signal, did not show any role in the intracellular trafficking of the 3a protein.

### The YXXΦ motif in 3a protein is responsible for its Golgi to plasma membrane sorting

**Figure 3:**
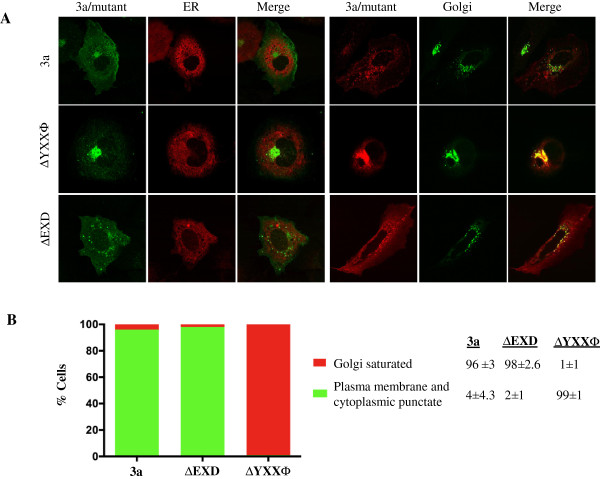
**The ∆YXXΦ mutant localizes to the Golgi compartment. (A)** Expression constructs for wild type 3a or its mutants were cotransfected with DsRed-ER or YFP-Golgi markers in Huh7 cells, followed by staining with anti-3a antibodies. **(B)** Quantitative estimates of subcellular distribution patterns of the 3a and its mutants. Golgi saturated and plasma membrane distribution pattern was quantified by looking at colocalisation with Golgi marker and surface staining respectively. A total of at least 50 different expressing cells were analyzed for each condition. Data shown are representative of three different experiments.

The subcellular distribution of the ΔYXXΦ protein was very different from that of the wild type 3a protein. To further characterize the compartments to which the mutant 3a proteins localize, we expressed them in Huh7 cells together with markers for the ER and Golgi compartments. The wild type and ΔEXD proteins were distributed to the cell surface and punctate structures that are likely to represent trafficking vesicles; these also showed minor colocalization with the Golgi compartment (Figure 3A). However, the ΔYXXΦ mutant protein was localized exclusively to the Golgi compartment (Figure 3A). Two subcellular distribution patterns were observed for the proteins. While the first consisted of a predominantly dispersed cytoplasmic and plasma membrane localization with a minor fraction of the protein in the Golgi compartment, the other showed a Golgi-saturated pattern. These distribution patterns for the proteins were quantified in multiple cells using the Golgi marker. An overwhelming majority of cells expressing wild type 3a or its ∆EXD mutant showed the first pattern, while all of the cells expressing the YXXΦ mutant showed the second pattern of localization (Figure 3B). The wild type 3a and ∆EXD proteins appear to be rapidly sorted from the Golgi complex to the plasma membrane. These results suggest that the di-acidic motif does not function as an ER export signal in this protein; if this was the case, the ∆EXD mutant protein would have been retained in the ER. None of the proteins showed any significant localization with the ER marker. The ΔYXXΦ mutant protein was retained in the Golgi complex but not in the ER region or at the plasma membrane, suggesting that the tyrosine-based sorting motif is crucial for the sorting of the 3a protein from Golgi to the plasma membrane.

### Increased Golgi retention of the 3a protein leads to increased targeting to lipid droplets

**Figure 4:**
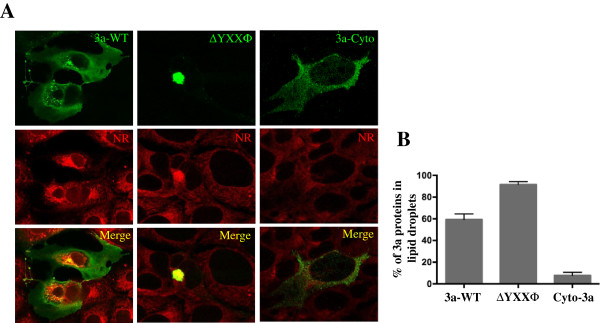
**The ∆YXXΦ mutant is targeted to lipid droplets. (A)** Huh7 cells were transfected with the indicated expression constructs. After 48 hr cells were treated with 1 mM fatty acids for 6 hr and then lipid droplets were stained with Nile Red. The 3a was stained with anti-3a antibodies. **(B)** Percent of the indicated 3a proteins in lipid droplets were quantified by determining colocalization coefficient in at least 50 different expressing cells as described. Data shown are representative of three different experiments.

Cytoplasmic lipid droplets have been proposed to act as storage vesicles for proteins [40]. Increased Golgi retention of Caveolin-2 is reported to result in targeting of the protein to lipid droplets [41]. We investigated whether this was also the case with Golgi-saturated 3a proteins. To visualize lipid droplets we used the specific dye Nile Red to stain transfected cells grown in culture media containing free fatty acids. On costaining with Nile Red, ~50% of the wild type 3a protein was found to be associated with lipid droplets, but the Cyto3a protein did not show any significant colocalization, indicating that the transmembrane region of the 3a protein was required for its targeting to lipid droplets (Figure 4). Presuming that these act as storage vesicles for excess or unfolded proteins, Golgi overload would cause increased targeting to lipid droplets. Expectedly, over 90% of the ΔYXXΦ mutant 3a protein localized to lipid droplets (Figure 4).

### Reduced levels of the ΔYXXΦ 3a protein are due to increased lysosomal degradation

**Figure 5:**
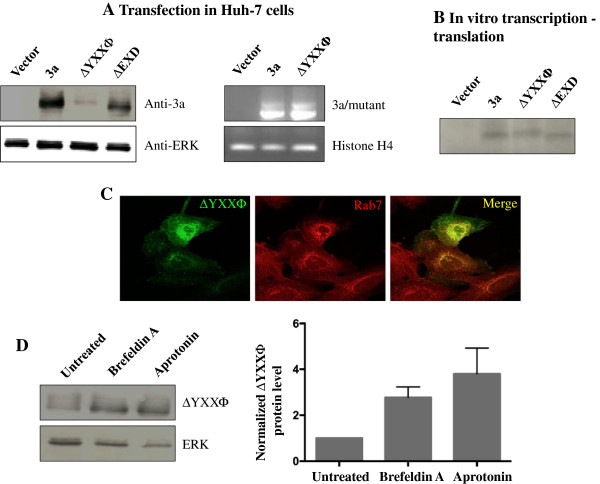
**Increased lipid droplet targeting is associated with increased lysosomal degradation. (A)** Expression levels of the wild type and mutant 3a proteins were evaluated 48 hrs post-transfection into Huh7 cells by western blotting with anti-3a antibodies and semi-quantitative RT-PCR with gene-specific primers. **(B)***In vitro* transcription-translation analysis of the wild type and mutant 3a proteins in a rabbit reticulocyte lysate system. **(C)** Colocalization of the ∆YXXΦ mutant protein and the late endosome/lysosomal marker Rab7. **(D)** Transfected Huh7 cells expressing the ∆YXXΦ mutant protein were treated with Brefeldin-A or Aprotinin for 12 hr before harvest. Western blotting was done with anti-3a and ERK (loading control) antibodies. Quantitation was done using Image-J software. Data shown are representative of two different experiments.

In multiple transient transfections, we observed that the ΔYXXΦ mutant protein was expressed at much lower levels as compared to the wild type 3a protein (Figure 5 A left panel). To investigate this, semi-quantitative RT-PCR was performed, which showed the mRNA levels of the wild type and mutant 3a proteins to be similar (Figure 5A right panel). This indicated that the mutant protein was either unstable or was actively degraded in the transfected cells. To explore this, we performed an *in vitro* transcription-translation experiment and found similar levels of the wild type and ΔYXXΦ mutant 3a proteins (Figure 5B), which supported the intracellular degradation model. Since the mutant protein was retained in lipid droplets, we hypothesized that this would lead to increased autophagy or lysosomal degradation. In fact, the ΔYXXΦ mutant protein localized with late endosome/lysosomal marker Rab7 (Figure 5C). To further confirm lysosomal degradation of the mutant protein, we used two inhibitors – Brefeldin A, which blocks ER-to-Golgi transport and Aprotinin A, which inhibits lysosomal proteases. Both of these led to 3 to 5 fold increased levels of the ΔYXXΦ mutant protein (Figure 5D). These findings clearly demonstrate that mutation of the tyrosine-based sorting motif causes abnormal trafficking of the 3a protein leading to increased accumulation in the Golgi compartment and lipid droplets and subsequent degradation in the lysosomal compartment.

## Discussion

The 3a protein is unique to SARS-CoV, not found in other human coronaviruses, and is important for disease pathogenesis. It functions as an ion channel [15], which is crucial for its pro-apoptotic role [42], and for this the 3a protein must traffic to the cell surface. This has been shown earlier [11] as well as in this report. However, the motifs or domains of the 3a protein required for its cell surface distribution have not been characterized.

In many proteins, a tyrosine based sorting motif is responsible for internalization of molecules from the plasma membrane to intracellular sites by specific recognition of the cargo containing this motif through the plasma membrane resident Clathrin AP-2 complex [21]. Consequently, it was proposed that the YXXΦ motif within the 3a protein would be important for its endocytosis [19]. If the YXXΦ motif were important for mediating endocytosis from plasma membrane, the mutant 3a protein lacking this motif would be retained in the plasma membrane. However, this mutant protein was not found in the plasma membrane, but in the Golgi compartment, suggesting a role for this motif in Golgi to plasma membrane transport. The YXXΦ motif is also recognized by the AP-1, AP-3 and AP-4 complexes, of which the AP-4 complex is responsible for Golgi to plasma membrane transport [22]. However, in a preliminary experiment we did not observe any interaction of the 3a protein with AP-4 in a yeast two-hybrid assay (unpublished data). This, as well as alternate mechanisms need further investigation. The di-acidic motif functions as a canonical ER export signal in many proteins [24-28]. A mutant 3a protein lacking this motif was efficiently transported from the ER to Golgi and the plasma membrane. Thus, the EXD motif does not functions as an ER export signal in the 3a protein.

We also found the 3a protein of SARS-CoV to be localized to lipid droplets. The transmembrane domains of the 3a protein are responsible for targeting it to lipid droplets since the Cyto3a protein that lacks the transmembrane domains did not localize to lipid droplets. It was shown earlier that hydrophobic domains of Perilipin A and the core proteins of HCV and GBV-B are crucial for lipid droplets targeting [35,39]. Lipid droplets are shown to act as storage vesicles for excess and unfolded proteins [34,40]. It has been reported that Golgi mislocalization of Caveolin-1 leads to its targeting into lipid bodies [43]. Over-expressed Caveolin-2 is also partially targeted to lipid droplets [38]. In addition Caveolin-1 accumulates in lipid droplets when it is linked to an ER-retrieval sequence (Cav-KKSL) [44]. A mutant Caveolin protein (Cav3^DGV^) that mislocalizes in the Golgi, accumulates irreversibly in lipid droplets [41]. We observed that the 3a ∆YXXΦ mutant that is retained in the Golgi is also associated with lipid droplets.

**Figure 6:**
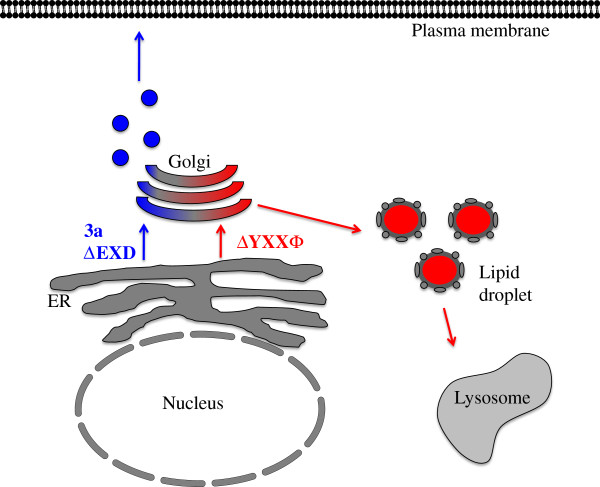
**A model of intracellular trafficking of the 3a protein.** The wild type 3a protein or the ∆EXD mutant are sorted from Golgi to plasma membrane whereas the ∆YXXΦ mutant protein is retained in the Golgi and is targeted to lysosomal compartment for degradation via lipid droplets.

Targeting to lipid droplets could be a pathway for protein degradation. In fact, inhibitors of autophagy and proteosomal degradation led to increased protein levels and lipid droplet association of Apolipoprotein B [37]. We observed that reduced protein levels of ∆YXXΦ mutant were associated with increased lysosomal degradation. There was a 3 to 5 fold increase in ∆YXXΦ protein levels on treatment with Brefeldin A or Aprotinin. Based on these data, we put forward a new model of intracellular distribution of the 3a protein (Figure 6). We propose that the wild type 3a protein is sorted efficiently from the ER and Golgi to the plasma membrane. The ∆YXXΦ mutant fails to reach the plasma membrane as it gets stuck in the Golgi compartment. This mutant protein is then targeted to lipid droplets and ultimately to the protein degradation machinery.

In conclusion, surface expression of the 3a protein is mediated by the crucial YXXΦ motif, in the absence of which the 3a protein is retained in the Golgi compartment destined for lysosomal degradation via lipid droplets. This finding demonstrates a novel role of the YXXΦ motif in intracellular protein transport.

## Materials and methods

### Materials

All common reagents were purchased from Sigma Chemical Co. (St. Louis, MO, USA) unless stated otherwise. COS-1 and Huh7 cells were obtained from American Type Culture Collection (Manassas, Va, USA), while MDCK and HT29 cells were obtained from the National Animal Cell Repository, National Centre for Cell Sciences (Pune, India). All cell lines were cultured at 37°C in 10% CO_2_ in complete Dulbecco modified Eagle medium (DMEM containing 1 g/lit glucose, 2 mM L-glutamine, 1.5 g/lit sodium bicarbonate, 0.1 mM non-essential amino acids, 0.1 mg/ml streptomycin, 100 U penicillin) and 5% fetal bovine serum (FBS). Anti-3a antibodies were generated using the purified cytoplasmic domain of the 3a protein as described earlier [29].

### Plasmid constructs

The *orf3a* (nucleotides 25,268 to 26,092) of the SARS-CoV genome (GenBank accession number NC_004718, Tor2 isolate) and its cytoplasmic domain were cloned into the eukaryotic plasmid vector pSGI-HA to give expression vectors pSGI-3A-HA and pSGI-3ACyto-HA, as described earlier [29]. N-terminal Myc-tag was added to the 3A and its different mutants by taking c-Myc-epitope tag from pGBKT7 vectors.

### Site directed mutagenesis

The ∆YXXΦ mutant of 3a was created by mutating the tyrosine and valine residues in the YXXV motif (aa 160–163) to alanine and glycine respectively, using primers YF1: TGTATACCAGCTAACAGCCTCACAG and YR1: CTGTGGCACTGTTAGCTGGTATACA. The ∆EXD mutant was created by mutating glutamic acid and aspartic acid residues in the EXD motif (aa 171–173) into alanines, using primers EF1: GTCGTTACTGCAGGTGCCGGCATTTC and ER1: GAAATGCCG GCACCTGCAGTAACGAC. Site directed mutagenesis was carried out using the QuickChange site directed mutagenesis kit system (Stratagene) according to the manufacturer’s protocol. All mutations were verified by DNA sequencing.

### Tranfection and western blotting

Cells were transfected using Lipofectin as described earlier [14] and were harvested in PBS 48 hr post-transfection. For experiments with inhibitors, transfected cells were treated with Brefeldin A or Aprotinin as per the manufacturer’s instruction (Sigma) for 12 hr before harvest. Western blotting was done after lysing the cells with lysis buffer (10 mM Tris pH7.6, 150 mM NaCl, 1% Triton-X100) with protease inhibitors as described earlier [14].

### Semi-quantitative RT-PCR

RNA was isolated from transfected cells 48 hr post-transfection using Trizol reagent (Invitrogen). Equal amounts of RNA were taken for reverse transcription using Superscript reverse transcriptase (Life Technologies). Semi-quantitative PCR was done using gene specific primers 3aF1, GAATTCATGGATTTGT TTATGAGATTT, and 3aR1, AGATCTCAAAGGCACGCTAGTAGT. The PCR reaction was carried out for only 20 cycles, which was found previously to be in the linear phase before reaching saturation.

### Immunofluorescence staining and subcellular localization

For transient transfection, cells were grown on coverslips to 40-50% confluence and transfected with Lipofectin reagent (Invitrogen, USA) in antibiotic-free and serum-free DMEM. Six hours post-transfection, the medium was removed and replaced with complete DMEM containing 5% FBS. Around 48 hr post-transfection, cells were washed with PBS and fixed with 2% paraformaldehyde for 15 min at room temperature. For antibody staining, cells were permeabilized with 100% methanol for 3 min at −20°C, blocked with PBS containing 5% goat serum for 45 min and then incubated with a 1:200 dilution of the primary antibodies in PBS for 1 hr at room temperature. The cells were washed thrice for 5 min each with PBS and then incubated with a 1:200 dilution of the relevant secondary antibodies conjugated with either Alexa 488 or Alexa 594 (Molecular Probes, USA) in PBS for 1 hr. The coverslips were mounted on slides with Antifade reagent (Bio-Rad, USA) and sealed with a synthetic rubber-based adhesive (Fevicol; Pidilite Industries, India). Confocal images were collected using a 60x objective in a Bio-Rad Radiance 2001 laser-scanning confocal system attached to a Nikon Eclipse TE-2000U inverted microscope. For subcellular localization, cells were cotransfected to express the required protein and a relevant fluorescent subcellular marker. For the studies reported here, the markers included DsRed-ER and YFP-Golgi that are part of the Living Colors™ Subcellular Localization Vector set (Clontech). To determine percent colocalization of different proteins; images were analyzed by Nikon Elements Software and the Pearson coefficient for colocalization was calculated.

### Nile red staining

Transfected Huh7 cells were transferred 48 hrs post-transfection to Glucose-free media (Sigma) containing 1 mM fatty acids (0.5 mM Oleic acid and 0.5 mM Palmitoleic acid). After 6 hr, the media was removed followed by a brief wash with PBS. Cells were stained with 10 μM Nile Red (Sigma) in PBS for 20 min at room temperature. The stain was removed and cells were washed twice with PBS. In case of co-staining with anti-3a antibodies, cells were first stained for 3a followed by Nile Red staining.

### In vitro transcription and translation

The gene to be expressed was cloned downstream of a T7 promoter in the pSGI expression vector [29]. The plasmid was then added to the reaction mixture containing T7 polymerase, amino-acid mixture (lacking methionine), appropriate buffer and rabbit reticulocyte lysate using the TNT® quick coupled transcription/translation system kit (Promega Corporation, USA) according to the manufacturer’s guidelines. Exogenously added [^35^S] methionine/cysteine was used (>1,000 Ci/ml) to label the protein as recommended for 90 min at 30°C. Subsequently, the *in vitro* synthesized proteins were analyzed by SDS-PAGE and fluorography.

## Competing interests

The authors have no competing interests.

## Authors’ contribution

K.P. conceived and designed the experiments; R.M. and K.P. performed the experiments and wrote the manuscript. Both authors read and approved the final manuscript.

## References

[B1] Drosten C Gunther S Preiser W van der Werf S Brodt HR Becker S Rabenau H Panning M Kolesnikova L Fouchier RA Berger A Burguiere AM Cinatl J Eickmann M Escriou N Grywna K Kramme S Manuguerra JC Muller S Rickerts V Sturmer M Vieth S Klenk HD Osterhaus AD Schmitz H Doerr HW Identification of a novel coronavirus in patients with severe acute respiratory syndrome N Engl J Med 2003 348 1967 1976 10.1056/NEJMoa030747 12690091

[B2] Lee N Hui D Wu A Chan P Cameron P Joynt GM Ahuja A Yung MY Leung CB To KF Lui SF Szeto CC Chung S Sung JJ Joynt GM Ahuja A Yung MY Leung CB To KF Lui SF Szeto CC Chung S Sung JJ A major outbreak of severe acute respiratory syndrome in Hong Kong N Engl J Med 2003 348 1986 1994 10.1056/NEJMoa030685 12682352

[B3] Poutanen SM Low DE Henry B Finkelstein S Rose D Green K Tellier R Draker R Adachi D Ayers M Chan AK Skowronski DM Salit I Simor AE Slutsky AS Doyle PW Krajden M Petric M Brunham RC McGeer AJ Identification of severe acute respiratory syndrome in Canada N Engl J Med 2003 348 1995 2005 10.1056/NEJMoa030634 12671061

[B4] Tsang KW Ho PL Ooi GC Yee WK Wang T Chan-Yeung M Lam WK Seto WH Yam LY Cheung TM Wong PC Lam B Ip MS Chan J Yuen KY Lai KN A cluster of cases of severe acute respiratory syndrome in Hong Kong N Engl J Med 2003 348 1977 1985 10.1056/NEJMoa030666 12671062

[B5] Marra MA Jones SJ Astell CR Holt RA Brooks-Wilson A Butterfield YS Khattra J Asano JK Barber SA Chan SY Cloutier A Coughlin SM Freeman D Girn N Griffith OL Leach SR Mayo M McDonald H Montgomery SB Pandoh PK Petrescu AS Robertson AG Schein JE Siddiqui A Smailus DE Stott JM Yang GS Plummer F Andonov A Artsob H The Genome sequence of the SARS-associated coronavirus Science 2003 300 1399 1404 10.1126/science.1085953 12730501

[B6] Rota PA Oberste MS Monroe SS Nix WA Campagnoli R Icenogle JP Penaranda S Bankamp B Maher K Chen MH Tong S Tamin A Lowe L Frace M DeRisi JL Chen Q Wang D Erdman DD Peret TC Burns C Ksiazek TG Rollin PE Sanchez A Liffick S Holloway B Limor J McCaustland K Olsen-Rasmussen M Fouchier R Gunther S Characterization of a novel coronavirus associated with severe acute respiratory syndrome Science 2003 300 1394 1399 10.1126/science.1085952 12730500

[B7] Yount B Roberts RS Sims AC Deming D Frieman MB Sparks J Denison MR Davis N Baric RS Severe acute respiratory syndrome coronavirus group-specific open reading frames encode nonessential functions for replication in cell cultures and mice J Virol 2005 79 14909 14922 10.1128/JVI.79.23.14909-14922.2005 16282490 PMC1287583

[B8] Tan YJ Goh PY Fielding BC Shen S Chou CF Fu JL Leong HN Leo YS Ooi EE Ling AE Lim SG Hong W Profiles of antibody responses against severe acute respiratory syndrome coronavirus recombinant proteins and their potential use as diagnostic markers Clin Diagn Lab Immunol 2004 11 362 371 15013989 10.1128/CDLI.11.2.362-371.2004 PMC371215

[B9] Ito N Mossel EC Narayanan K Popov VL Huang C Inoue T Peters CJ Makino S Severe acute respiratory syndrome coronavirus 3a protein is a viral structural protein J Virol 2005 79 3182 3186 10.1128/JVI.79.5.3182-3186.2005 15709039 PMC548460

[B10] Shen S Lin PS Chao YC Zhang A Yang X Lim SG Hong W Tan YJ The severe acute respiratory syndrome coronavirus 3a is a novel structural protein Biochem Biophys Res Commun 2005 330 286 292 10.1016/j.bbrc.2005.02.153 15781262 PMC7092867

[B11] Tan YJ Teng E Shen S Tan TH Goh PY Fielding BC Ooi EE Tan HC Lim SG Hong W A novel severe acute respiratory syndrome coronavirus protein, U274, is transported to the cell surface and undergoes endocytosis J Virol 2004 78 6723 6734 10.1128/JVI.78.13.6723-6734.2004 15194747 PMC421683

[B12] Freundt EC Yu L Goldsmith CS Welsh S Cheng A Yount B Liu W Frieman MB Buchholz UJ Screaton GR Lippincott-Schwartz J Zaki SR Xu XN Baric RS Subbarao K Lenardo MJ The open reading frame 3a protein of severe acute respiratory syndrome-associated coronavirus promotes membrane rearrangement and cell death J Virol 2010 84 1097 1109 10.1128/JVI.01662-09 19889773 PMC2798367

[B13] Law PT Wong CH Au TC Chuck CP Kong SK Chan PK To KF Lo AW Chan JY Suen YK Chan HY Fung KP Waye MM Sung JJ Lo YM Tsui SK The 3a protein of severe acute respiratory syndrome-associated coronavirus induces apoptosis in Vero E6 cells J General Virol 2005 86 1921 1930 10.1099/vir.0.80813-0 15958670

[B14] Padhan K Minakshi R Towheed MA Jameel S Severe acute respiratory syndrome coronavirus 3a protein activates the mitochondrial death pathway through p38 MAP kinase activation J Gen Virol 2008 89 1960 1969 10.1099/vir.0.83665-0 18632968

[B15] Lu W Zheng BJ Xu K Schwarz W Du L Wong CK Chen J Duan S Deubel V Sun B Severe acute respiratory syndrome-associated coronavirus 3a protein forms an ion channel and modulates virus release Proc Natl Acad Sci U S A 2006 103 12540 12545 10.1073/pnas.0605402103 16894145 PMC1567914

[B16] Obitsu S Ahmed N Nishitsuji H Hasegawa A Nakahama K Morita I Nishigaki K Hayashi T Masuda T Kannagi M Potential enhancement of osteoclastogenesis by severe acute respiratory syndrome coronavirus 3a/X1 protein Arch Virol 2009 154 1457 1464 10.1007/s00705-009-0472-z 19685004 PMC7086770

[B17] Minakshi R Padhan K Rani M Khan N Ahmad F Jameel S The SARS Coronavirus 3a protein causes endoplasmic reticulum stress and induces ligand-independent downregulation of the type 1 interferon receptor PLoS One 2009 4 e8342 10.1371/journal.pone.0008342 20020050 PMC2791231

[B18] Zeng R Yang RF Shi MD Jiang MR Xie YH Ruan HQ Jiang XS Shi L Zhou H Zhang L Wu XD Lin Y Ji YY Xiong L Jin Y Dai EH Wang XY Si BY Wang J Wang HX Wang CE Gan YH Li YC Cao JT Zuo JP Shan SF Xie E Chen SH Jiang ZQ Zhang X Characterization of the 3a protein of SARS-associated coronavirus in infected vero E6 cells and SARS patients J Mol Biol 2004 341 271 279 10.1016/j.jmb.2004.06.016 15312778 PMC7127270

[B19] Tan YJ The Severe Acute Respiratory Syndrome (SARS)-coronavirus 3a protein may function as a modulator of the trafficking properties of the spike protein Virol J 2005 2 5 10.1186/1743-422X-2-5 15703085 PMC549520

[B20] Huang C Narayanan K Ito N Peters CJ Makino S Severe acute respiratory syndrome coronavirus 3a protein is released in membranous structures from 3a protein-expressing cells and infected cells J Virol 2006 80 210 217 10.1128/JVI.80.1.210-217.2006 16352545 PMC1317539

[B21] Bonifacino JS Dell'Angelica EC Molecular bases for the recognition of tyrosine-based sorting signals J Cell Biol 1999 145 923 926 10.1083/jcb.145.5.923 10352010 PMC2133128

[B22] Bonifacino JS Traub LM Signals for sorting of transmembrane proteins to endosomes and lysosomes Annu Rev Biochem 2003 72 395 447 10.1146/annurev.biochem.72.121801.161800 12651740

[B23] Letourneur F Klausner RD A novel di-leucine motif and a tyrosine-based motif independently mediate lysosomal targeting and endocytosis of CD3 chains Cell 1992 69 1143 1157 10.1016/0092-8674(92)90636-Q 1535555

[B24] Hanton SL Renna L Bortolotti LE Chatre L Stefano G Brandizzi F Diacidic motifs influence the export of transmembrane proteins from the endoplasmic reticulum in plant cells Plant Cell 2005 17 3081 3093 10.1105/tpc.105.034900 16214902 PMC1276031

[B25] Phillipson BA Pimpl P daSilva LL Crofts AJ Taylor JP Movafeghi A Robinson DG Denecke J Secretory bulk flow of soluble proteins is efficient and COPII dependent Plant Cell 2001 13 2005 2020 10.1105/tpc.13.9.2005 11549760 PMC139448

[B26] Nishimura N Balch WE A di-acidic signal required for selective export from the endoplasmic reticulum Science 1997 277 556 558 10.1126/science.277.5325.556 9228004

[B27] Votsmeier C Gallwitz D An acidic sequence of a putative yeast Golgi membrane protein binds COPII and facilitates ER export EMBO J 2001 20 6742 6750 10.1093/emboj/20.23.6742 11726510 PMC125768

[B28] Mikosch M Hurst AC Hertel B Homann U Diacidic motif is required for efficient transport of the K + channel KAT1 to the plasma membrane Plant Physiol 2006 142 923 930 10.1104/pp.106.087064 16950859 PMC1630742

[B29] Padhan K Tanwar C Hussain A Hui PY Lee MY Cheung CY Peiris JS Jameel S Severe acute respiratory syndrome coronavirus Orf3a protein interacts with caveolin J Gen Virol 2007 88 3067 3077 10.1099/vir.0.82856-0 17947532

[B30] Murata M Peranen J Schreiner R Wieland F Kurzchalia TV Simons K VIP21/caveolin is a cholesterol-binding protein Proc Natl Acad Sci U S A 1995 92 10339 10343 10.1073/pnas.92.22.10339 7479780 PMC40792

[B31] Cohen AW Razani B Schubert W Williams TM Wang XB Iyengar P Brasaemle DL Scherer PE Lisanti MP Role of caveolin-1 in the modulation of lipolysis and lipid droplet formation Diabetes 2004 53 1261 1270 10.2337/diabetes.53.5.1261 15111495

[B32] Rothberg KG Heuser JE Donzell WC Ying YS Glenney JR Anderson RG Caveolin, a protein component of caveolae membrane coats Cell 1992 68 673 682 10.1016/0092-8674(92)90143-Z 1739974

[B33] Martin S Parton RG Lipid droplets: a unified view of a dynamic organelle Nat Rev Mol Cell Biol 2006 7 373 378 10.1038/nrm1912 16550215

[B34] Cermelli S Guo Y Gross SP Welte MA The lipid-droplet proteome reveals that droplets are a protein-storage depot Current biol CB 2006 16 1783 1795 10.1016/j.cub.2006.07.062 16979555

[B35] Subramanian V Garcia A Sekowski A Brasaemle DL Hydrophobic sequences target and anchor perilipin A to lipid droplets J Lipid Res 2004 45 1983 1991 10.1194/jlr.M400291-JLR200 15342676

[B36] Jiang H He J Pu S Tang C Xu G Heat shock protein 70 is translocated to lipid droplets in rat adipocytes upon heat stimulation Biochim Biophys Acta 2007 1771 66 74 10.1016/j.bbalip.2006.10.004 17175194

[B37] Ohsaki Y Cheng J Fujita A Tokumoto T Fujimoto T Cytoplasmic lipid droplets are sites of convergence of proteasomal and autophagic degradation of apolipoprotein B Mol Biol Cell 2006 17 2674 2683 10.1091/mbc.E05-07-0659 16597703 PMC1474802

[B38] Fujimoto T Kogo H Ishiguro K Tauchi K Nomura R Caveolin-2 is targeted to lipid droplets, a new "membrane domain" in the cell J Cell Biol 2001 152 1079 1085 10.1083/jcb.152.5.1079 11238462 PMC2198803

[B39] Hope RG Murphy DJ McLauchlan J The domains required to direct core proteins of hepatitis C virus and GB virus-B to lipid droplets share common features with plant oleosin proteins J Biol chem 2002 277 4261 4270 10.1074/jbc.M108798200 11706032

[B40] Welte MA Proteins under new management: lipid droplets deliver Trends Cell Biol 2007 17 363 369 10.1016/j.tcb.2007.06.004 17766117

[B41] Pol A Luetterforst R Lindsay M Heino S Ikonen E Parton RG A caveolin dominant negative mutant associates with lipid bodies and induces intracellular cholesterol imbalance J Cell Biol 2001 152 1057 1070 10.1083/jcb.152.5.1057 11238460 PMC2198820

[B42] Chan CM Tsoi H Chan WM Zhai S Wong CO Yao X Chan WY Tsui SK Chan HY The ion channel activity of the SARS-coronavirus 3a protein is linked to its pro-apoptotic function Int J Biochem Cell Biol 2009 41 2232 2239 10.1016/j.biocel.2009.04.019 19398035 PMC7108357

[B43] Wyse BD Prior IA Qian H Morrow IC Nixon S Muncke C Kurzchalia TV Thomas WG Parton RG Hancock JF Caveolin interacts with the angiotensin II type 1 receptor during exocytic transport but not at the plasma membrane J Biol Chem 2003 278 23738 23746 10.1074/jbc.M212892200 12692121

[B44] Ostermeyer AG Paci JM Zeng Y Lublin DM Munro S Brown DA Accumulation of caveolin in the endoplasmic reticulum redirects the protein to lipid storage droplets J Cell Biol 2001 152 1071 1078 10.1083/jcb.152.5.1071 11238461 PMC2198801

